# A novel perspective on bone tumors: advances in organoid research

**DOI:** 10.3389/fphar.2025.1550163

**Published:** 2025-04-08

**Authors:** Zebing Ma, Yibing Liu, Rui Chen, Huayu Fan, Liang Kong, Xiangyang Cao

**Affiliations:** ^1^ Hunan University of Chinese Medicine, Changsha, Hunan, China; ^2^ Luoyang Orthopedic Hospital of Henan Province (Orthopedic Hospital of Henan Province), Zhengzhou, Henan, China; ^3^ Institute of Intelligent Medical and Bioengineering Henan Academy of Traditional Chinese Medicine Sciences, Zhengzhou, Henan, China; ^4^ Henan Province Artificial Intelligence Engineering Research Center for Bone Injury Rehabilitation, Luoyang Orthopedic Hospital of Henan Province (Orthopedic Hospital of Henan Province), Zhengzhou, Henan, China; ^5^ Henan University of Chinese Medicine, Zhengzhou, Henan, China

**Keywords:** bone tumor, organoid, 3D culture, drug development, precision medicine

## Abstract

Bone tumor organoids are three-dimensional cell culture models derived from patient tissues or cells, capable of highly replicating the growth patterns and cell interactions of bone tumors *in vitro*. Current treatments for bone tumors are hindered by challenges such as drug resistance, recurrence, and metastasis. Organoids enhance the physiological relevance of bone tumor models, thereby improving treatment precision and overcoming the limitations of current therapeutic approaches. Organoid technology has made preliminary applications in bone tumor research, including primary bone tumors, metastatic bone tumors, and bone marrow-derived bone tumors. This review will explore the establishment of bone tumor organoids, summarize their applications and prospects in various bone tumor diseases, and discuss their integration with emerging technologies. Additionally, the limitations and future directions of bone tumor organoid research will be discussed. In the future, bone tumor organoids are expected to promote the further development of precision medicine.

## 1 Introduction

Bone tumors are typically classified as benign or malignant, with the latter posing significant clinical concern (Choi and Ro, 2021). Malignant bone tumors (hereafter referred to as bone tumors) are further categorized into primary and metastatic types. Primary bone tumors, such as osteosarcoma (OS), Ewing sarcoma (ES), and chondrosarcoma (CS), predominantly affect younger individuals and are characterized by high malignancy and metastatic potential (Xi et al., 2023). In contrast, metastatic bone tumors are frequently observed in patients with advanced-stage cancer, especially those with breast cancer (BCa), prostate cancer (PCa), and lung cancer (LCa) (Coleman et al., 2020).

Organoids are three-dimensional (3D) tissue models, derived from *in vitro* culture systems, that recapitulate key structural and functional characteristics of their corresponding organs or tissues. Under appropriate culture environment, organoids demonstrate self-organization, forming cellular compositions and spatial structures, as well as performing physiological functions similar to the native organs. The concept of organoids was first introduced by Sato et al., who successfully cultured small intestinal stem cells to generate crypt-like structures (Sato et al., 2011). With continuous advancements in organoid research, including the development of brain, liver, and lung organoids (Xu et al., 2022), these models have demonstrated great potential for application in simulating tumor microenvironments (TME), tumor metastasis studies, and drug screening.

While traditional models have shown great potential in the treatment of bone tumors, their inherent limitations hinder the development of novel therapeutic approaches. For instance, while animal models can partially simulate the growth and metastasis of bone tumors, interspecies differences often lead to clinical failure of some therapeutics. Two-dimensional (2D) cell culture techniques are limited due to the absence of tissue structure and TME, as they consist of homogeneous cell populations proliferating in a flat surface. Consequently, these models fail to capture the inherent heterogeneity among tumor cells (Duval et al., 2017). To address this challenge, Sung et al. developed the first 3D co-culture system incorporating human MG-63 or HS27A cells with C4-2. Compared with traditional culture dishes, 3D co-culture systems effectively promoted sustained morphological alterations in bone matrix cells (MG63 or HS271 cells) (Sung et al., 2008). Unlike conventional cell lines, patient-derived (PD) tissues or cells preserve the unique genomic and functional heterogeneity of the original tissue (Lee et al., 2020). In oncology research, spheroids were the first 3D models developed; however, spheroids derived from single cell lines lack the structural complexity necessary to accurately replicate the architecture of the native tissues. In contrast, organoids represent advanced 3D models capable of replicating various biological characteristics of the original tissue, such as differentiation, extracellular matrix formation, and the expression of specific functional properties (Azar et al., 2021).

Advanced research in bone tumors has enabled the development of 3D TME, in both synthetic and natural matrices, which closely replicate the growth patterns of bone tumors, intercellular interactions, and their relationship with the matrix under physiological conditions. OS organoids have been successfully established as preclinical therapeutic models, facilitating high-throughput drug screening and personalized treatment through single-cell sequencing (Huang B. et al., 2024). This review will explore the establishment of bone tumor organoids, summarize their applications and prospects in various bone tumor diseases, and discuss their integration with emerging technologies. Additionally, the limitations and future directions of bone tumor organoid research will be discussed.

## 2 Establishment of bone tumor organoids

**FIGURE 1:**
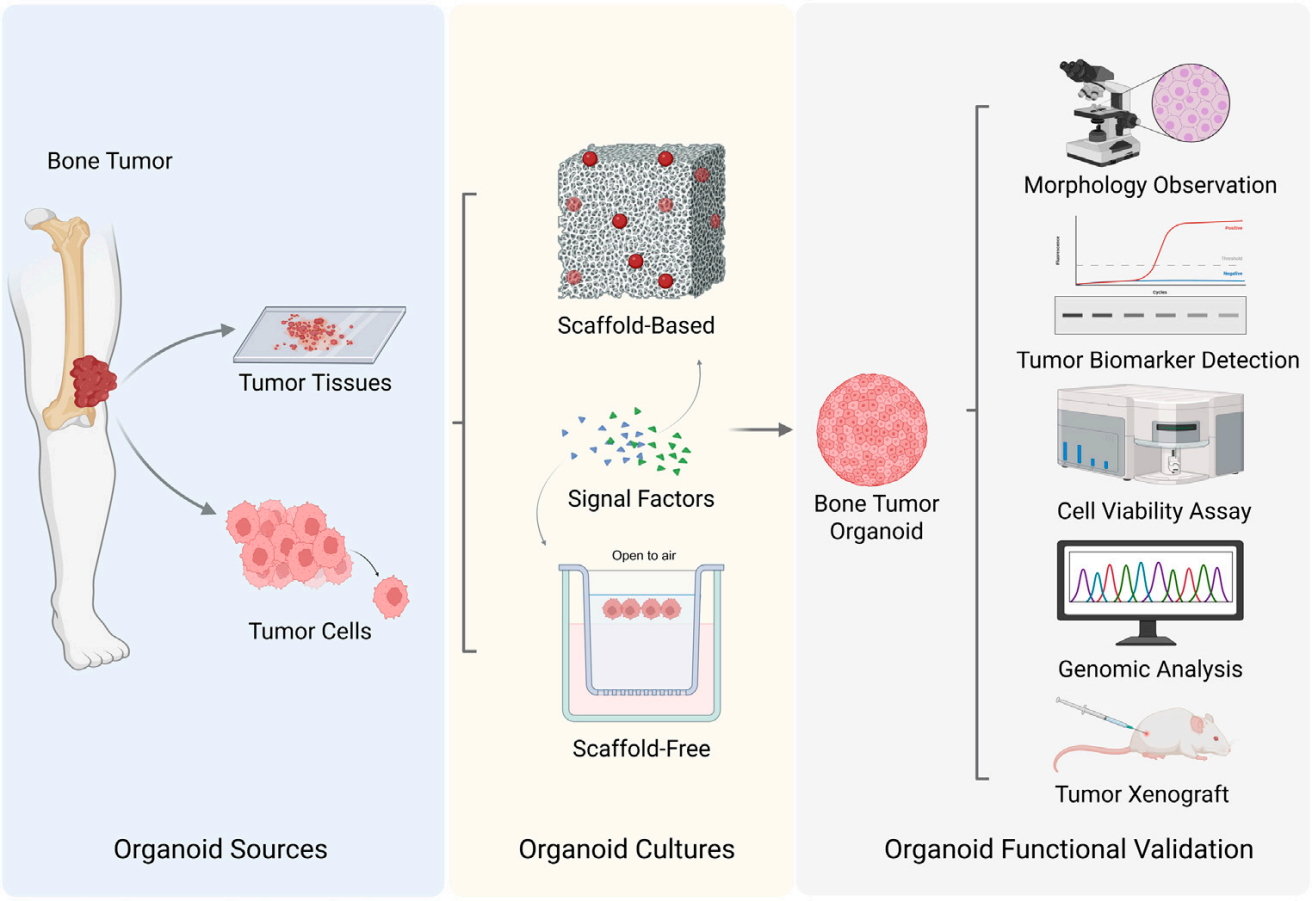
Strategies for bone tumor organoids establishment (Created with BioRender.com).

Several key steps are involved in the development of bone tumor organoids. The most important step is the selection of the appropriate tissue or organ source. Next, the selection of an appropriate culture scaffold, whether composed of natural matrices or synthetic materials, is a critical step. This is followed by the establishment of tissue structures through optimized culture conditions and advanced technical methodologies. The final step involves the assessment of the success rate or stability of the generated bone tumor organoids. Currently, research on bone tumor organoids is in the exploratory phase, with bone tumor organoid subtypes having been successfully developed. Figure 1 illustrates the various key steps involved in the process of establishing bone tumor organoids and their subtypes. Table 1 summarizes the culture characteristics of different types and sources of bone tumor organoids.

**TABLE 1:** Analysis of culture characteristics of different bone tumor organoids.

Diseases	Tissue or cell source	Expansion medium	Formation time	Assay method	Success rate	References
OS	Tissues from 24 patients (20 biopsies, 12 surgeries)	DMEM/F12, penicillin, streptomycin, nystatin, nicotinamide, N-acetylcysteine, B-27 minus vitamin A, EGF, Rspo1, SB-202190, Y-27632, Noggin	2 weeks	HE staining, ICC, Biomarker SOX9	95% (biopsies) 91.7% (surgeries)	Nie et al. (2022)
OS	Tissues from 2 patients	DMEM/F12, penicillin, streptomycin, nystatin, nicotinamide, N-acetylcysteine, B-27 minus vitamin A, EGF, Rspo1, SB-202190, Y-27632, Noggin	2 weeks	ICC, PDX	non-quantitative data	Zhang et al. (2024)
CMA	Tissues from 24 patients	coated microchambers (composition not reported)	72 hours	Cell morphology observation, ICC	non-quantitative data	Scognamiglio et al. (2019)
CMA	Tissues from 7 patients	MammoCult (composition not reported)	5 days	Cell morphology observation, HE Ki-67 and brachyury staining	100%	Al Shihabi et al. (2022)
CMA	Tissues from 5 patients	DMEM/F12, GlutaMax, B27, nicotinaminde, N-acetyl cysteine, HEPES, Wnt3a, Noggin, Rspo1, FGF-10, EGF, A83-01, Y-27632	5 days	HE and brachyury staining, Exome sequencing	90.75%–99.08%	Xu et al. (2023)
ES	Tissues from 1 patient	IMDM (composition not reported)	3 weeks	Cell morphology observation, Surface maker CD99, Genotype analysis, STR analysis	99%	Maurer et al. (2022)
MGCTB	Tissues from 1 patient	DMEM/F12, HEPES, GlutaMAX, nicotinamide, N-acetylcysteine, B-27, EGF, gastrin I, Noggin, Rspo1, SB-202190, Afamin/Wnt3a	6 days	Cell morphology observation, HE staining, IHC	60%–80%	Suzuki et al. (2023)
BCa BoM	Tissues from 1 patient	Not reported	Not reported	Single-cell RNA sequencing	non-quantitative data	Ding et al. (2022)
PCa BoM	Tissues from 5 patients	Advanced D-MEM/F-12, Primocin, GlutaMAX, HEPES, Y-27632, penicillin/streptomycin, nicotinamide, Rspo1, N-acetylcysteine, B-27, SB-202190, Noggin, DHT, HGF, EGF, FGF-10, FGF2, PGE_2_, A83-01	7 days	IHC, Flow cytometry	non-quantitative data	La Mana et al. (2020)
LCa BoM	Tissues from 5 patients	Advanced D-MEM/F-12, HEPES, Glutamine, A83-01, B-27, EGF. Noggin, Rspo1, SB-202190, Wnt3a	10 days	HE staining, IHC	100%	He et al. (2020)
LCa BoM	Tissues from 18 patients	Advanced D-MEM/F-12, GlutaMax, B27, N2, Y-27632, FGF-10, EGF, N-acetylcysteine, Noggin, A83-01	2-3 weeks	HE staining, IHC, Transcriptomics analysis	77.8%	Hu et al. (2024)
OS	OS single-cell suspension from gene knockout rats	D-MEM/F-12, EGF, FGF2, FGF10, Rspo1, Noggin, A38-01, Y-27632	6 days	HE staining, IHC	non-quantitative data	Wang et al. (2024)
CS	CS cell line, HACs	HG-DMEM, penicillin/streptomycin, ciprofloxacin, amphotericin	Not reported	Cell morphology observation, RTCA, IHC	non-quantitative data	Veys et al. (2021)
PCa BoM	Human PCa cell line VCaP, mPOBs	MEMα, penicillin/streptomycin, (composition not reported)	Not reported	Cell morphology observation, ICC	non-quantitative data	Thomas et al. (2022)
LCa BoM	LCa BoM single-cell suspension from patients	Advanced D-MEM/F-12 (composition not reported)	Not reported	HE staining	lower	He et al. (2020)
MM	Human MM cell line, BMSCs, EPCs	RPMI 1640, GlutaMax, penicillin/streptomycin (composition not reported)	2 weeks	Cell morphology observation, ICC	non-quantitative data	Braham et al. (2018)
MM	BMSCs, Human MM cell line	Complete bone culture medium (composition not reported), RANKL, M-CSF	3 weeks	RTCA, Alcian blue staining, Transcriptomics analysis	non-quantitative data	Visconti et al. (2021)

Note: OS, osteosarcoma; EGF, epidermal growth factor; HE, hematoxylin and eosin; ICC, immunocytochemistry; PDX, Patient-derived xenograft; CMA, chordoma; FGF, fibroblast growth factor; ES, ewing sarcoma; MGCTB, malignant giant cell tumor of bone; IHC, immunohistochemistry; BCa, Breast cancer; BoM, bone metastases; PCa, Prostate cancer; DHT, dihydrotestosterone; HGF, hepatocyte growth factor; PGE_2_, Prostaglandin E_2_; LCa, Lung cancer; HAC, human articular chondrocytes; RTCA, Real-time cell analysis; mPOB, murine preosteoblast cell line; MM, multiple myeloma; BMSCs, Bone marrow mesenchymal stem cells; EPCs, Endothelial progenitor cells; RANKL, Receptor activator of nuclear factor-κB ligand; M-CSF, Macrophage colony-stimulating factor.

### 2.1 Organoid sources

#### 2.1.1 Tissue sources

Bone tumor organoids derived from patient tissue biopsies undergo an initial processing step involving mechanical dissociation and enzymatic digestion, yielding tissue clumps referred to as organoid seeds. Under specific 3D culture conditions and with appropriate supplementation of growth factors, these cells undergo self-organization, forming organoids that partially replicate the structural and functional properties of the original tissue. Although the initial dissociation process results in partial loss of TME components, the organoid cells retain patient-specific characteristics, including gene expression profiles, genetic mutations, and cellular morphology (Yoshida, 2020).

Primary bone tumor organoids are derived from the initial tumor site and are typically obtained directly from patient biopsy samples or surgically resected tissues, including those from OS, chordoma (CMA), and ES. Currently, research has significantly focused on OS. Nie used 20 biopsy tissues and 12 surgical samples of OS patients to develop OS organoids, with success rates of 95% and 91.7%, respectively. Notably, these tissue-derived OS organoids formed within 2 weeks and continued to proliferate with stable phenotypes for several months (Nie et al., 2022). Further research using advanced methods successfully developed OS organoids from two patient samples, with cell proliferation and immunofluorescence (IF) assays further validating the formation success rate and growth characteristics of these organoids (Zhang et al., 2024a). CMA presents significant research challenges due to its rarity, thus organoid models derived from patient tissues are vital for a deeper understanding of the disease mechanism (Walcott et al., 2012). Researchers successfully generated 80–200 μm organoids from tissues of 24 CMA patients to explore their growth characteristics (Scognamiglio et al., 2019). Additionally, organoid cultures were established using CMA tissues from various anatomical locations and disease stages, with subsequent quantification analysis of organoid area changes revealing strong proliferative capacity (Al Shihabi et al., 2022). Another challenge in organoid establishment involves ES, which exhibits high cellular heterogeneity and genetic rearrangements. Maurer established organoids from biopsy tissue of a single ES patient; cell labeling and biomarker analysis further validating the feasibility of establishing tissue-derived organoids for ES (Maurer et al., 2022). Giant cell tumors of bone, a highly benign sub type of bone tumors, exhibit limited availability in their active samples compared to other bone tumors. Consequently, researchers could only develop malignant giant cell tumor of bone (MGCTB) organoids from biopsy tissue of a single patient, with proliferation assays showing an organoid survival rate between 60% and 80% (Suzuki et al., 2023).

For metastatic bone tumors, the tissue source is typically selected from the metastatic lesion site rather than the primary tumor site. Tissues from metastatic sites more accurately model the interaction between metastatic bone tumors and the bone microenvironment, rather than the biological behavior of primary tumors. BCa bone metastasis (BoM) is often osteolytic and predominantly manifest in advanced disease stages. The scarcity of PD samples is attributed to complications associated with chemotherapy, as well as ethical and logistical challenges in obtaining fresh tissue samples. Subsequently, only one study has reported culturing organoids from tissue specimens collected from a BCa patient’s left pelvis and right tibia (Ding et al., 2022). In contrast, tissue collection in PCa BoM is often easier since it primarily manifests as osteoblastic metastasis, necessitating earlier diagnosis through imaging techniques such as bone scans. In 2014, for the first time, researchers cultured organoids from biopsy tissue of 18 PCa BoM patients, selecting two cases (L2 lumbar vertebra metastasis and acetabulum metastasis) for further culturing over 6 months (Gao et al., 2014). Based on this foundation, subsequent studies established organoid models from tissue samples of five PCa BoM patients, gradually developing an extensive organoid repository necessary for studying the highly heterogeneous disease (La Manna et al., 2020). LCa BoM which predominantly affects the spine, occurring in 90% of cases, exhibits osteolytic metastasis characteristics leading to vertebral destruction and symptoms such as thoracic back pain and lower limb neurological disorders (Hu et al., 2022). Spinal surgery, which is the direct method for symptom relief, provides a significant opportunity to obtain tissue samples. Researchers cultured organoids from surgical tissue of five LCa BoM patients. The organoids exhibited irregular morphology and positive SOX9 expression, a marker for OS, within 10 days of culture (He et al., 2020). Hu’s study further confirmed the feasibility of culturing tissue-derived organoids, with the results indicating a culture success rate of 77.8% from 18 cases of LCa BoM (Hu et al., 2024).

#### 2.1.2 Cell sources

Cell-derived organoids are developed from a single cell type or line. Commonly used cell sources include tumor cell lines, stem cells, or specific osteoblasts (such as osteoblast cell lines or bone marrow stromal cells). Under specific culture media, these cells depend on 3D scaffolds to rebuilding structures and functions similar to those of the original tissue. The controllable nature of cell-derived organoids enables researchers to precisely regulate cellular composition and population dynamics. Furthermore, cell lines represent stable populations that have undergone long-term culture and passaging, resulting in greater consistency in organoid formation and experimental outcomes, while mitigating limitations associated with sample availability.

In studies involving primary bone tumor organoids, cell-derived organoids can be directly adjusted at the cellular level using modern gene editing technologies, compared to tissue-derived organoids. For example, primary OS derived from animal models with double knockouts of *RB1* and *TP53*, or triple knockouts of *p53*, *Rb1*, and *SK2*, was dissociated into single-cell suspensions and expanded into organoid cultures. OS organoids formed within 6 days (Wang et al., 2024). In establishing CS organoids, the SW1353 CS cell line is often used for culture, since the cartilage lacks blood vessels and has a greater matrix composition. In a study, Veys cultured the SW1353 and human articular chondrocytes within collagen scaffolds and analyzed cell cycle progression after 72 h (Veys et al., 2021). Despite these advancements in the development of organoids for primary bone tumor, no research has shown cell-derived organoid establishment for CMA, ES, or MGCTB.

In metastatic bone tumors studies, the cell source is typically derived from the metastatic cell lines from the primary sites. During metastasis, tumor cells undergo various biological changes, including enhanced invasiveness, migration ability, and adaptability to various microenvironments, along with genetic and phenotypic alterations. Therefore, using cell lines from the primary site closely represents the early dynamics of tumor metastasis, exhibiting stronger metastatic potential. In BCa BoM, the MDA-MB-231 cell line is highly metastatic. Baldassarri et al. incorporated MDA-MB-231 cells into an engineered bone marrow (eBM) derived from bone marrow-derived mesenchymal stem cells (BMSCs); however, the study did not assess the impact of other cell types involved in extracellular matrix remodeling on BCa BoM (Baldassarri et al., 2024). In PCa BoM research, bone marrow-derived epithelial organoids were initially established, then tumor cell lines (LNCaP, VCaP, and PC3) were introduced to form co-culture systems. Thomas et al. improved this technique by developing heterotypic organoids consisting of VCaP and mouse osteoblast precursor cell line MC3T3-E1, where the inner layer consisted of osteoblast precursor cells and the outer layer by PCa cells (Thomas et al., 2022). Additionally, in the single study on LCa BoM organoids, researchers prepared single-cell suspensions from PD OS lung metastatic tissue, demonstrating the feasibility of single-cell organoid culture from metastatic tissue, although only a few single cells formed organoid structures. Existing research suggests that single cells are more suited for primary OS culture (He et al., 2020).

Bone marrow-derived bone tumors, such as multiple myeloma (MM), closely interact with surrounding bone marrow cells (including bone marrow stromal cells and immune cells). Notably, due to the heterogeneity of these tumors, it is challenging to fully represent all tumor characteristics using tissue samples only. Moreover, primary MM cells have limited proliferative capacity *in vitro*. Subsequently, when selecting cell lines for 3D culture, researchers often opt for cell lines that can be easily manipulated. Reagan et al. established the first co-culture model of human-derived MM cell line (MMIS) and BMSCs; however, their 3D model focused on cell interactions in the bone marrow microenvironment, rather than on traditional bone tumor organoids (Reagan et al., 2014). Research indicates that MM alters the bone marrow microenvironment, producing abnormal mesenchymal stem cells (MSCs) and vasculature that support tumor growth (Vacca et al., 2014). Subsequently, Braham co-cultured MM cell lines (OPM2), CD138^+^, MSCs, and endothelial progenitor cells (EPCs) in varying ratios, forming blood vessel-like structures in the 3D culture. The MSCs changed their phenotype *in vitro* to resemble that of the patient’s MM, thereby indicating a successful formation of MM organoid model (Braham et al., 2018). Visconti further improved upon this finding by generating eBM using BMSCs to form normal bone-like fragments, then co-culturing them with different MM cell lines (MMIS, NCI-H929, and U266B1), inducing bone resorption and lytic lesions to closely replicate the bone lesions associated with MM (Visconti et al., 2021).

Induced pluripotent stem cells (iPSCs) exhibit potential for unlimited proliferation and multi-lineage differentiation. Early researchers reprogrammed PD iPSCs and induced them under specific conditions to differentiate into osteoblast precursors. Osteogenesis-inducing factors were incorporated to further promote differentiation into MSCs and osteoblasts, facilitating the generation of OS *in vitro* from iPSCs derived from Li-Fraumeni syndrome patients (Lee et al., 2015). iPSC-derived MSCs, upon directed differentiation, enhanced OS invasiveness through the secretion of bioactive factors. However, their tumor-promoting effects in the TME were less pronounced compared to BMSCs (Zhao et al., 2015). In recent studies, researchers have developed eBM from iPSC-derived MSCs, human umbilical vein endothelial cells (HUVECs), and cord blood hematopoietic stem/progenitor cells to facilitate the generation of BCa BoM organoids (Baldassarri et al., 2024). Although some studies indicate that iPSCs can differentiate into cartilage and osteoblast lineages to facilitate the establishment of cartilage organoids and complex bone marrow-like organoids (O'Connor et al., 2021; Frenz-Wiessner et al., 2024), their specific role in bone tumor organoids requires further investigation.

#### 2.1.3 Limitations of sources

Tissue sources limitations include challenges in obtaining tissue samples, tissue heterogeneity, culture challenges, and ethical and legal constraints. Notably, core regions of bone tumors may be hypoxic and necrotic, while peripheral areas are more proliferative (Cao et al., 2015). Subsequently, the tissue samples obtained may only represent specific regions of the bone tumor and fail to capture the tumor’s heterogeneity and biological variations between the regions. Multi-region sampling, including sampling from various regions of the pelvis, L3 vertebra, and thoracic vertebra in the same CMA patient, has been employed to generate organoids (Al Shihabi et al., 2022). This approach partially overcomes tissue source limitations. Additionally, during tumor cell proliferation, the accumulation of genetic variants leads to cells acquiring different adaptive characteristics (Bridge et al., 1999). Tissue-derived bone tumor organoids represent the genetic background of a specific time point, thereby lacking the ability to capture the genetic drift that occurs over time. This limitation constrains their capacity to accurately model tumor evolution and dynamic genetic alterations. This limitation can be addressed by periodically changing samples and conducting multi-timepoint sampling, such as collecting samples before and after bone tumor treatment at different stages of disease progression.

Although cell-derived organoids can be established in a short period and used for research purposes, they are characterized by various limitations which include loss of original tumor characteristics, absence of TME, limited proliferation capacity, and difficulty in extracellular matrix (ECM) reconstruction. Single cells cannot fully represent the heterogeneity of bone tumor cells or accurately replicate the complex TME (such as interactions among osteoblasts and osteoclasts, among others). For instance, in BoM, some cells exhibit active proliferative state, while others exhibit dormant or metastatic potential (Clézardin et al., 2021). Subsequently, multicellular co-culture (involving cells such as osteoblasts and MSCs) has been used to optimize bone microenvironment in the development of PCa BoM organoids (Thomas et al., 2022). Additionally, developmental bias is another critical issue in cell-derived organoids. Development bias can lead to overexpression of specific cell populations, such as osteoblasts, resulting in excessive matrix deposition and fibrosis in organoids, altering tumor cell proliferation and failing to accurately model the balance of cell populations in the original tumor. To address developmental bias, researchers have optimized the culture by adjusting the ratios of MSCs, EPCs, and MM cells to avoid genetic drift during a 28-day culture period (Braham et al., 2018). Moreover, compared to tissue-derived organoids, cell-derived organoids lack ECM support, leading to structural and functional differences compared to the original tumor tissues. Future research may simulate the mechanical environment of bone tissue using mechanical stimulation (such as fluid shear stress) to promote ECM reconstruction (Ozcivici et al., 2010).

### 2.2 Supportive matrix

#### 2.2.1 Natural matrix materials

Natural matrix materials are derived from biological organisms. They include substances such as Matrigel (Laperrousaz et al., 2018), collagen, gelatin, and alginates, derived from basement membranes of mouse kidney cells. These materials provide physicochemical properties similar to those of the *in vivo* environment and support cell adhesion, proliferation, and tissue structure formation through binding sites on their surfaces (Hughes et al., 2010). For example, complexes of gelatin and collagen can closely model the natural bone structure (Sharifi et al., 2016). Natural matrix materials not only provide structural support, but also closely simulate the bone TME, facilitating the transmission of biochemical signals (Zhang et al., 2010). Additionally, they exhibit good biocompatibility (Yan et al., 1994), which reduces immune responses and cytotoxicity, allowing the organoid seeds to remain stable during *in vitro* culture.

#### 2.2.2 Synthetic scaffold materials

Synthetic scaffold materials are engineered from artificially synthesized polymers, including poly (lactic-co-glycolic acid) (PLGA), polycaprolactone (PCL), and polyurethane. Synthetic scaffold materials allow for precise control over their physical properties, such as porosity and adjustable mechanical performance, enabling them to simulate the bone microstructure, compared to the natural matrix materials. Researchers have used PCL, composed of hydroxyapatite and barium titanate, to model bone tissue porosity (35%–45%) and stiffness (40–55 MPa) to conduct studies involving BCa BoM (Shah et al., 2022). Complex 3D structures can be formed using 3D printing or self-assembly methods, providing space for tumor cell growth and supporting organoid tissue structure formation. Additionally, synthetic materials can introduce bioactive molecules through chemical modifications or functionalization to promote tumor cell adhesion. For instance, researchers have seeded MSCs onto polyurethane scaffolds to promote osteogenic differentiation, thereby establishing a bone matrix for OS cell culture (Contessi Negrini et al., 2022). Additionally, MSCs have been cultured on PCL scaffolds for 12 days to design a 3D niche tailored for ES (Molina et al., 2020). Also, synthetic scaffold materials facilitate controlled drug release. For example, PLGA scaffolds are commonly used as drug delivery systems to assess the long-term effects of drugs on bone tumor organoids, depending on the research aims (Qiu and Liu, 2023). However, in simple experiments (such as suspension cultures or exploration of bone tumor cells *in situ*), scaffold materials may not be necessary, since such examination depends on the self-organizing ability of the bone tumor cells. However, in simple experiments (such as suspension cultures or exploration of bone tumor cells *in situ*), scaffold materials may not be necessary.

There are distinct differences between natural matrix materials and synthetic scaffold materials in their ability to replicate the TME. Natural matrices primarily focus on biological simulation, showing superior efficacy in replicating interactions between tumor cells and the extracellular matrix. Contrastingly, synthetic scaffolds effectively replicate the mechanical environment by modifying surface properties, such as functionalized polymer coatings, to enhance biological performance. In terms of experimental duration, natural matrix materials are well-suited for short-term organoid cultures due to their biodegradability and biocompatibility, which facilitate the rapid establishment of microenvironments conducive for cell growth and differentiation. In contrast, synthetic scaffold materials, characterized by their high stability and adjustable porosity, are more suitable for long-term organoid cultures. Regarding clinical relevance, natural matrix materials, though less versatile in application, are advantageous for early-stage tumor research, drug screening, and investigations into metastasis mechanisms. In contrast, synthetic scaffold materials offer greater customization in terms of their shape and mechanical properties, facilitating patient-specific tumor modeling. Their adaptability makes them particularly valuable in applications such as simulating bone structure invasion and facilitating bone tissue repair.

### 2.3 Culture conditions and techniques

The typical culture environment for bone tumor organoids includes a temperature of 37°C, an atmospheric composition of 95% air and 5% CO_2_, 95%–100% humidity level, and a physiological pH of 7.2–7.4. However, some culture situations may require a hypoxic environment with 1%–5% O_2_ and a lower pH (4.0–6.5) to simulate the TME (Veys et al., 2021; Deng et al., 2023). In most studies, the basal culture medium is DMEM, RPMI-1640, or MEM, providing the essential cell growth nutrients. Growth factors such as epidermal growth factor (EGF), B-27 minus vitamin A, fibroblast growth factor, and insulin-like growth factor are incorporated to promote the proliferation of bone tumor cells. Supplements like FBS or other serum substitutes are used to provide nutritional support, while antibiotics or antifungals like penicillin, streptomycin, and amphotericin are used to maintain a healthy and disease-free culture environment. Antioxidants such as niacinamide and N-acetylcysteine help to clear intracellular free radicals, protecting cells from oxidative stress. Regulators of pH including buffers like HEPES and bicarbonates are used to maintain a stable culture media environment.

The culture conditions for various bone tumor organoids primarily depend on the incorporated cytokines. For primary bone tumors, such as OS and CMA, signal pathway modulators are often added to regulate the balance between bone differentiation and proliferation. For example, in the Wnt/β-catenin pathway, modulators like RSPO1, p38 mitogen-activated protein kinase (MAPK) inhibitor SB-202190, Rho-associated protein kinase inhibitor Y-27632, transforming growth factor beta (TGF-β) inhibitor A83-01, and bone morphogenetic protein inhibitors like Noggin are commonly used (Nie et al., 2022; Wang et al., 2024; Xu et al., 2023). Similar culture conditions are used for metastatic bone tumors. Some studies suggest that adding Wnt-3A does not significantly improve the generation of BCa organoids; however, excessively high concentrations of EGF and SB-202190 may inhibit the efficiency of organoid formation (Sachs et al., 2018). To simulate tumor invasion and metastasis processes, researchers add the A83-01 is often added to suppress excessive TGF-β signaling, preventing it from promoting tumor growth, invasion, and immune evasion in PCa BoM (Karkampouna et al., 2021). Furthermore, when establishing MM organoids, the addition of the cytokine, receptor activator of nuclear factor kappa-B ligand (RANKL), facilitates the generation of osteoclasts, while the inclusion of macrophage colony-stimulating factor (M-CSF) synergistically functions with RANKL to support the bone resorption process (Visconti et al., 2021; Anloague et al., 2024).

Cultivation techniques applied to bone tumor organoids involve the use of air-liquid interface (ALI) culture. This technique involves the placement of tumor cells at the interface between culture medium and the air to achieve a more realistic cell-matrix interactions (Wakamatsu et al., 2022). Using this technique, researchers observed that MGCTB organoids showed a 3.3-fold increase in vitality after 72 h in ALI culture. However, the organoids exhibited morphological distortion, weak cell adhesion, and defects in passage due to breakage (Suzuki et al., 2023). The 3D matrix culture, a common method for bone tumor organoid cultivation, uses natural or synthetic matrix materials to facilitate the growth of cells in a 3D space and form structures that closely replicate the *in vivo* conditions. This technique is often used for drug sensitivity testing and preclinical evaluation. However, the use of 3D matrix culture is associated with challenges such as difficulty in precisely quantifying cell morphology and proliferation rates, as well as the complexity of managing large and complex datasets generated during high-throughput screening, typically characterize the use of 3D matrix culture. Subsequently, microcarrier culture, which provides a larger surface area to support higher cell density, is often used to address such limitation, thereby allowing for multidrug resistance testing. McNeill et al. observed that osteogenically enhanced human MSCs and OS cells interacted via bridging attachment points, simulating the dynamic balance between osteogenesis and osteolysis (Mcneill et al., 2018). While microcarriers have a great potential to resolve drug resistance, they lack the natural tissue structure and fail to accurately model the complex bone matrix environment and the invasiveness or metastatic ability of bone tumors. Bioreactors, which provide for controlled conditions like temperature, oxygen, and interstitial fluid flow, can offer more physiologically accurate cell culture environments than static culture systems, thereby enhancing cell proliferation and differentiation efficiency. Under flow culture conditions, hMSCs exhibited a more flattened morphology, while fluid shear stress upregulated αvβ3 integrin and MMP-9 expression, thereby promoting PCa BoM. However, at high flow rates, TGF-β1 expression increased, promoting apoptosis through the Smad signaling pathway and inhibiting tumor cell growth (Jasuja et al., 2023). These findings highlight the necessity for precise adjustment of the flow rate of the bioreactor to optimize experimental outcomes. Hanging drop culture, which uses gravity to allow OS cells to suspend in liquid droplets to form micro-tissues, is a low-cost and easy-to-operate system suitable for small-volume organoid culture and early tumor research (Rimann et al., 2014). The Rotary cell culture system simulates the *in vivo* 3D environment and provides flowing liquid, promoting nutrient and oxygen exchange inside and outside organoids, overcoming the limitations of static cultures. In a 3D co-culture model of MM cells integrating BMSCs and HUVECs, the system enhanced MM cell survival, maintained stable expression of markers such as CD138 and light chains, and elevated IL-6 and Ang-2 concentrations, thereby closely modeling the TME cell-matrix interactions. However, this system requires more time and increased costs for the assessment of cell proliferation and changes in drug resistance (Belloni et al., 2018).

### 2.4 Functional validation

The functional validation of bone tumor organoids establishment involves a series of systematic evaluations. First, morphological observation and phenotypic assessment are conducted to determine whether the organoids exhibit the basic morphology and histological characteristics of bone tumors. This assessment is achieved through microscopy techniques including optical microscopy, fluorescence microscopy, scanning electron microscopy, in combination with histological staining methods such as H&E staining, Masson trichrome staining (for TME matrix evaluation), and bone calcium staining (for assessing bone matrix deposition), to observe the organoid structure (Visconti et al., 2021). Second, tumor marker expression is analyzed to confirm the biological identity of the organoids. Commonly used methods include immunohistochemistry, IF staining, real-time PCR, and Western blotting. During OS organoid establishment, key biomarkers such as SOX9 and vimentin, as well as stem cell markers like CD133 and GPC3, were well-maintained (Nie et al., 2022). Additionally, IF staining is used to detect Ki-67 and cleaved Caspase-3 to quantify cellular proliferation rate within OS organoids (Zhang X. et al., 2024). Third, flow cytometry and CCK-8 assays are used to evaluate cell proliferation and apoptosis in the organoids. Flow cytometry analysis revealed that two miRNAs increased sub-G1 phase events in the CS cell line, SW1353, inducing apoptosis (Veys et al., 2021). Fourth, transwell migration assays are used to assess the ability of organoids to simulate the invasion and metastasis of bone tumors. For instance, under varying concentrations of CCL5, transwell assays demonstrated a significant increase in CMA cell migration, confirming the invasiveness of the organoids (Xu et al., 2023). Fifth, genomic and epigenetic analysis, specifically RNA-seq, is conducted to compare gene expression patterns between bone tumor organoids and normal bone tissue or other tumor subtypes, allowing for the identification of tumor-specific molecular signatures. Protein chip analysis further facilitates the characterization of tumor cell properties, including proliferation, invasion, and differentiation. Additionally, metabolomic analysis is used to assess metabolic changes in organoids and therapeutic responses within the organoid models. Lastly, xenograft experiments are used to validate the reliability and effectiveness of organoids to provide a foundation for further research. By subcutaneously implanting OS organoids into mice models, the proliferative capacity of the organoids is assessed from the perspective of tumor dynamics (Nie et al., 2022). Additionally, genetically modified mouse tumor models (such as knockout or oncogene-activated models) are used to compare organoid-derived tumors with primary OS, ensuring that the organoids accurately replicate the histological characteristics of primary bone tumors (Wang et al., 2024).

### 2.5 Standardization challenges

Although bone tumor organoids hold significant clinical potential, their inherent complexity introduces significant variability in reproducibility and consistency across different laboratory settings. First, the heterogeneity of cell sources directly affects the formation of organoid models. Therefore, selecting and standardizing tumor cells or stem cells populations from diverse origins is crucial to ensuring the reliable and consistent expression of key biological characteristics within organoid models. In BoM research, tissue sources for organoids must have at least 10% tumor content and exhibit the ability to continuously proliferate for over 6 months (Gao et al., 2014). Second, bone tumor organoid culture involves the use of various growth factors, matrices, and medium components, such as synthetic scaffold with specific mechanical properties. However, substantial variability in these factors across different laboratories can significantly impact organoid development, structural integrity, and functional outcomes. To ensure reproducibility, standardized protocols should be established for matrix components, cell density, and other factors. Third, a standardized functional validation protocol for bone tumor organoids should be developed. While researchers have determined the success rate of establishing bone tumor organoid, most studies lack quantitative processes, relying instead on microscopic or fluorescent staining for assessment. Given these issues, standardized protocols for bone tumor organoids are under development, with an open, shared platform being established to regularly control the quality of cell sources and culture conditions. These efforts are aimed at gradually overcoming standardization challenges.

## 3 Application of organoids in bone tumors

**FIGURE 2:**
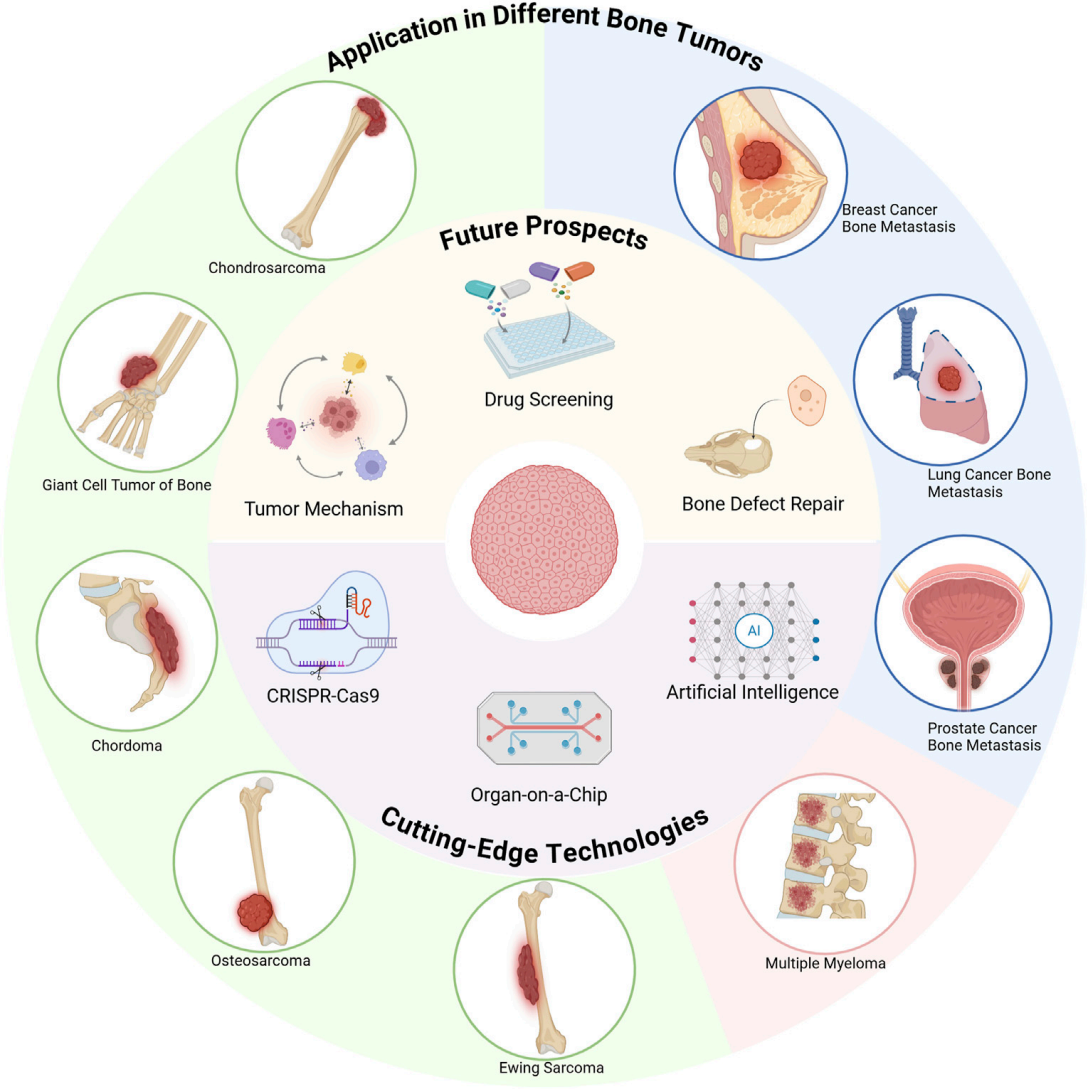
Innovative applications and emerging technologies of bone tumor organoids (Created with BioRender.com).

Bone tumor organoids offer a highly representative *in vitro* model for accurately simulating the growth and development characteristics of bone tumors. Depending on the tumor source and case-specific characteristics, organoids can be further classified into distinct subtypes (Figure 2). Although research in bone tumor organoids remains in its early exploratory stage, existing studies have demonstrated the substantial potential of bone tumor organoids in both basic research and clinical applications.

### 3.1 Primary malignant bone tumors

Currently, OS is the primary area of research in tumor organoids. Notably, OS is the most common primary bone tumors, predominantly arising in the metaphyseal region of long bones in young individuals (Meltzer and Helman, 2021). Recent studies have explored the use of a multicell-type lung organoid model to recreate an *in vivo* TME for OS cells, independent of the LCa BoM model. Additionally, researchers have investigated the therapeutic potential of the STAT5 inhibitor, pimozide, which targets the JAK/STAT signaling pathway (Nelson et al., 2012), downregulating OS-associated cell markers such as DCLK1, CD44, and CD133. This process results in the reduction of the OS stem cell growth, and inhibiting 3D bone sphere formation (Subramaniam et al., 2020). This indicates that organoids can be used to test the efficacy of pimozide on OS. Due to the intra-tumor heterogeneity of OS, no specific effective targets have been identified apart from the conventional chemotherapy. In 2022, Nie identified mutations in the *GPC3* gene in OS. Subsequent studies demonstrated that organoids could more accurately simulate the tumor responses, establishing an optimal *in vitro* platform for GPC3-targeted therapies. Future studies could use GPC3-positive OS-derived organoids as preclinical treatment models to screen OS patients with heightened sensitivity to GPC3-targeted treatment, thereby facilitating personalized therapeutic strategies (Nie et al., 2022). However, the effectiveness of different *GPC3* levels and its interaction with the immune system in OS organoids still requires further research. Similarly, OS organoids provide a more precise model for investigating the impact of genetic alterations, such as *RB1* and *TP53* deletions, on OS pathophysiology. These organoids serve as valuable preclinical models for assessing the anti-tumor efficacy of SKP2 inhibitors (Zhang et al., 2024a), and for validating the expression patterns of PDGFD and PDGFRB, as well as the spatial distribution and binding characteristics of ligand-receptor interactions (Huang Y. et al., 2024). Additionally, kinase inhibitors have emerged as potential treatments for OS, however, due to the complex nature of OS pathogenesis, single-agent kinase inhibitors have exhibited suboptimal clinical efficacy. Recent studies have shown that testing and screening various drug combinations using OS organoids validated the synergistic effects of ALK and FLT3 inhibitors in inhibiting OS growth (Zhang et al., 2024a).

CS is the second most prevalent bone tumor, primarily affecting adults between the ages of 30–70. It primarily affects the long bones of the limbs, pelvis, and scapula (Nazeri et al., 2018). Characterized by a dense cartilage matrix and limited blood supply, CS exhibits poor responsiveness to chemotherapy, especially under hypoxic conditions, and demonstrates intrinsic resistance to radiotherapy (Onishi et al., 2011). Given its resistance to traditional treatments, researchers have started exploring the application of organoids in CS studies. Veys et al. were the first to use CS organoids to validate the effects of miRNA on SW1353 CS cells. The results indicated that miR-342-5p could induce cell death in both normoxic and hypoxic environments, with a more pronounced effect under hypoxia (Veys et al., 2021). However, due to the intrinsic heterogeneity of CS, as exemplified by the LB35 cell line’s lack of significant response to miRNA treatment, further studies are required to evaluate the *in vivo* efficacy and safety of miRNA-based therapies. Conclusively, organoid models provide a crucial experimental platform for future CS treatment research, particularly in evaluating new therapeutic strategies within specific tumor microenvironmental conditions such as hypoxia.

ES is a highly aggressive bone tumor that predominantly affects children and adolescents, with a high recurrence rate and metastatic potential leading to poor overall survival outcomes. Genetic analysis indicates that 10%–13% of ES patients carry pathogenic mutations in DNA damage repair genes, suggesting that DNA damage-targeting drugs may provide new treatment options for ES (Brohl et al., 2017). Maurer used an organoid model to study the impact of *BARD1* deletion on ES cell sensitivity to DNA damage. Using the PSaRC318 cell line as a representative model for *BARD1* mutations, the study demonstrated that PARP inhibitors (such as Talazoparib and Niraparib) in combination with radiation therapy, showed therapeutic efficacy. These findings highlight potential treatment options for ES patients with similar genetic mutations (Maurer et al., 2022). However, due to the rarity of ES, research in this area remains limited, necessitating further studies to validate these therapeutic approaches.

CMA originates from embryonic remnants of the notochord and commonly affects anatomical regions such as the skull base, sacrococcygeal region, and spinal column (Karpathiou et al., 2020). Due to CMA complex anatomical location, complete surgical resection is challenging (Fuchs et al., 2005), and traditional drug treatments are ineffective (Stacchiotti and Sommer, 2015). Consequently, the development of PD CMA organoid models has emerged as a promising approach for personalized drug screening and therapeutic development. Researchers have successfully established CMA organoids that accurately recreate the TME, while preserving key immune components like CD8^+^ lymphocytes and PD-L1^+^ cells. This model has been used to evaluate the efficacy of the immune drug nivolumab, accelerating drug screening for this slow-growing tumor (Scognamiglio et al., 2019). Furthermore, chimeric antigen receptor T-cell (CAR-T) immunotherapy, an emerging treatment method that uses genetically engineered T-cells to recognize and attack tumor cells, has shown potential in CMA immune therapy. B7–H3, a highly expressed antigen on CMA cells, has been identified as an optimal target for CAR-T therapy. Data indicate that introducing IL-7 into B7-H3 CAR-7 cells (B7-H3 CAR-7/IL-7), may effectively kill CMA cells in the organoid model and exhibit sustained anti-tumor effects (Wu et al., 2024a). Additionally, VEGFR/TGF-βscFv CAR-T cells exhibited significant anti-tumor effects against CMA cells (Wu et al., 2024b), providing new research directions and clinical application prospects for CMA immunotherapy. Moreover, by establishing multiple patient-specific CMA organoids, researchers have observed distinct growth patterns correlated with clinical characteristics, further underscoring the utility of organoid models in capturing tumor heterogeneity and advancing personalized treatment strategies (Al Shihabi et al., 2022).

Giant cell tumor of bone originates from mesenchymal tissues within the bone marrow and predominantly affects females between the ages of 20 and 50. It primarily arises in the epiphyses of the long bones and progressively invades the metaphysis (Liu et al., 2021). While giant cell tumor of bone is benign, malignant transformation has been observed in recent years. Due to their high recurrence rate and malignant transformation potential, MGCTB has become a key focus in bone tumor research. Researchers have established MGCTB organoid models derived from representative clinical cases. These organoids exhibited features similar to clinical tumors, such as chemotherapy resistance and high expression of genes like *CD44* and *TWIST*, which are often associated with tumor progression and metastasis (Suzuki et al., 2023). Additionally, genomics-based therapeutic interventions in these models have provided valuable insights into potential treatment strategies for MGCTB, reinforcing the role of organoid systems as preclinical platforms for evaluating novel therapeutic approaches.

### 3.2 Metastatic bone tumors

Metastatic bone tumors are cancer cells that spread from primary tumors to the bones through the blood or lymphatic system. During metastasis, tumor cells exhibit complex biological characteristics that further exacerbate the treatment of BoM. Currently, organoids have been used in 3D culture systems to simulate the TME, providing a new platform for exploring tumor-cell interactions with surrounding cells. Research on BoM organoids has experienced significant progress in BCa, LCa, and PCa studies, while studies on other types of BoM are still limited. Subsequently, this section focuses on organoid applications in these three tumor types.

BCa is a highly invasive malignant tumor with a strong propensity for hematogenous and lymphatic dissemination, frequently leading to skeletal metastases. BCa BoM typically presents as osteolytic metastasis, with disseminated tumor cells finding suitable microenvironments for survival, thereby damaging bone tissues such as the spine, ribs, and femur (Leone et al., 2017). To elucidate the molecular mechanism and therapeutic response of BCa BoM, researchers have employed organoid models that replicate key features of the disease. These organoid models have demonstrated the ability to preserve the somatic mutation characteristics of BCa BoM, including clinically relevant genetic alterations such as PI3K^E545K^ and BRCA1^D1834H^. Additionally, organoids demonstrated sensitivity to specific treatments, particularly strong growth inhibition with alpelisib and talazoparib. These findings suggest that organoid models help in examining genetic mutation features of BCa BoM, and can also be used for drug screening and personalized treatment (Ding et al., 2022). Furthermore, organoid models have provided critical insights into the molecular mechanisms of BCa BoM. For instance, research has revealed that SOST enhances the activity of the STAT3/TGF-β/KRAS signaling pathway, promoting BoM proliferation. Experimental investigations showed that the SOST-STAT3 axis using the SOST-neutralizing antibody S6 significantly inhibits BCa BoM progression, providing theoretical support for its clinical potential utility (Sun et al., 2022). To further enhance the physiological relevance of BCa BoM models, researchers transplanted PD organoids into an eBM model, successfully recreating patient-specific tumor-immune interactions. For example, this model revealed changes in the bone marrow niche, such as biased bone marrow generation, increased granulocyte numbers, and tumor cell immune evasion mechanisms (Baldassarri et al., 2024). These findings not only advance the understanding of BCa BoM immune microenvironment dynamics but also provide new directions for exploring tumor cell interactions and developing targeted therapeutic strategies.

The mechanism of LCa BoM typically involves cancer cells invading the bones through blood or lymph flow. Subsequently, they modify the BoM environment to promote cancer cell growth. Especially small cell LCa and adenocarcinoma, often exhibit osteolytic metastatic phenotype, with preferential dissemination to the spine, hip, ribs, and pelvis (Cheng et al., 2023a; Cheng et al., 2023b). Organoid models have emerged as important tools for studying the pathophysiological mechanism of LCa BoM, predicting treatment responses, and facilitating screening new drugs. Studies have shown that lung metastatic OS organoids preserve the original morphology of the tumor and tissue features, as well as respond to immune checkpoint inhibitors by reactivating CD8^+^ T cell cytotoxic function, increasing the proportion of CD8^+^/CD4^+^ T cells in the total CD3^+^ T cell population (He et al., 2020). This immunomodulatory effect enhances antitumor immune responses and has significantly contributed to the advancement of immunotherapeutic strategies targeting lung metastases in OS. Further research has highlighted the important role of organoids in LCa BoM. Comparative analyses have established that organoids closely resemble the genetic and phenotypic features of original tumors, including resistance to *EGFR* mutation-related TKIs. In a study conducted by Hu et al., treatment with DMAb led to the downregulation of RANKL expression in organoids. Transcriptomic analysis further revealed that DMAb significantly affected the biological behavior of LCa BoM by regulating multiple signaling pathways, such as TNF, cAMP, and PI3K (Hu et al., 2024). These findings highlight novel therapeutic targets and potential treatment strategies for LCa BoM. Moreover, organoid-based studies have provided mechanistic insights into ceRNA network regulation in LCa BoM, especially implicating the XLOC_006941/hsa-miR-543/NPRL3 axis. Additionally, research has demonstrated the involvement of GATA3 and Th2 cells in shaping the TME, contributing to tumor progression and immune evasion. The integration of organoids with targeted therapy has led to the identification of E7449 and dupilumab, as well as inhibitors of organoid proliferation (Liu X. et al., 2025), providing new methods to improve the prognosis and clinical outcomes of LCa BoM patients.

PCa exhibits a strong osteoblastic metastatic phenotype, characterized by excessive bone formation rather than the osteolytic bone degradation commonly observed in BCa LCa metastases. Metastatic dissemination preferentially occurs in regions of the skeletal system with high vascularization, including the spine, pelvis, and femur (Zhang, 2019). Early investigation utilizing 3D co-culture system comprising PCa cells (C4-2), OS MG63 cells, and human osteoblast cells, revealed that bone matrix cells underwent permanent morphological changes, indicating that 3D microenvironmental conditions accelerate cancer growth and metastasis (Sung et al., 2008). Building on these findings, organoid models have been used to replicate PCa cell colonization in BoM niches, evaluating the adhesion, migration, and colony formation of PCa metastatic cells in bone microenvironments enriched with Tenascin-C. Experimental evidence indicates that inhibiting the interaction between integrin α9β1 and tenascin-C may serve as a potential therapeutic strategy for PCa BoM (San Martin et al., 2017). These findings reveal the impact of the bone microenvironment in shaping metastatic behavior and highlight the potential intervention points for therapeutic development. Organoids studies have further validated the efficacy of multiple treatment strategies targeting PCa BoM, including mTOR inhibitors such as Rapalink-1, targeted drugs like disulfiram, and standard treatments like abiraterone and enzalutamide. Additionally, the assessment of cancer stem cell populations expressing CD44^+^ and ALDH^+^ markers has provided insights into the differential responsiveness of PCa-BoM to various treatment regimens (La Manna et al., 2020). Additionally, organoid models were used as preclinical models to study tumor resistance mechanisms, particularly under androgen pathway-directed therapy, revealing new castration-resistant mechanisms in PCa BoM cells (Lee et al., 2022). Building on previous research, scientists have recently developed heterotypic organoids composed of osteoblast precursors and PCa cells, which effectively replicate the TME and reveal how extracellular matrix glycoprotein Tenascin-C affects treatment resistance by regulating AR-V7’s post-translational stability. Additionally, investigations into the effects of various hormonal conditions (such as testosterone, estradiol, and enzalutamide) on treatment responses, have provided novel perspectives for understanding PCa BoM treatment responses (Thomas et al., 2022).

### 3.3 Bone marrow-derived malignant tumors

Bone marrow-derived malignancies, primarily originating from malignant plasma cells within the bone marrow cavity, encompass MM and plasmacytomas. These tumors are characterized by their disruption of bone marrow hematopoiesis and their associations with bone destruction (Rodriguez, 2021). To elucidate the pathological mechanisms underlying these processes, various research groups have used organoid models. Researchers used a 3D bone marrow microenvironment model to simulate organoid structures, validating the detrimental effects of MM cells on MSC osteogenic differentiation. Notably, the results indicated that upregulating miR-199a-5p restored the osteogenic ability in MM-MSC co-culture systems, offering new insights into MM BoM and bone metabolism disorders (Reagan et al., 2014). Building on these findings, Visconti et al. developed MM organoid models to study the dynamic changes between normal bone homeostasis and bone destruction processes. Their study further delineated the mechanisms of drug resistance in MM, specifically via the WNT signaling pathway, and evaluated the effectiveness of immune modulators, monoclonal antibodies, and bisphosphonates in restoring bone metabolic balance (Visconti et al., 2021). These findings underscore the potential of organoids as a platform for investigating MM-associated drug resistance and therapeutic screening. Subsequent studies used organoid models for drug or gene silencing to inhibit the expression of CLPP. Suppression of this protein in triggered endoplasmic reticulum stress and apoptosis, leading to reduced organoid growth, decreased cell viability, and disruption of the cell cycle. These findings offer new directions for MM treatment, especially in developing drugs targeting CLPP (Qin et al., 2025). Additionally, Martini et al. developed a 3D skeletal micro physiological system to support the long-term survival of primary plasma cells and evaluated CAR-T cell immunotherapy targeting plasma cells (Martini et al., 2024). Although this study did not specifically focus on MM, it demonstrated the potential application of CAR-T therapy in plasma cell malignancies. In summary, the application of organoid models in MM research underscores their significant potential in simulating disease microenvironments, unveiling molecular mechanisms, and evaluating therapeutic efficacy. These findings provide new research platforms and directions for the treatment of bone marrow-derived tumors.

## 4 Innovations of bone tumor organoids

### 4.1 Elucidating tumor matrix interactions

In the study of the tumor-matrix interaction mechanisms, organoids serve as an *ex vivo* dynamic model, enabling the detailed examination of how tumor cells alter the composition of the extracellular matrix through the secretion of enzymes, growth factors, and other bioactive molecules. These matrix components, including collagen, hyaluronic acid, and fibronectin, play crucial roles in tumor growth, metastasis, and drug resistance formation. Existing studies indicate that intricate interactions between bone tumor cells, osteoblasts, osteoclasts, and immune cells actively remodel the local tumor microenvironment, thereby influencing tumor cell behavior and progression. For example, overexpression of the bone resorption factor RANKL promotes epithelial-mesenchymal transition (EMT) in PCa cells, while integrin α2 binding with collagen in the bone matrix helps tumor cells attach to bone tissue and migrate into the bone by remodeling the cytoskeleton (Ziaee and Chung, 2014).

Moreover, advancements in single-cell RNA sequencing technology have facilitated a more detailed characterization of the transcriptional features of distinct cell subpopulations within bone tumors. This approach has provided critical insights into the functional heterogeneity of the TME. For instance, tumor-associated fibroblasts can modulate matrix stiffness by secreting cytokines, thereby promoting tumor cell proliferation and migration (Högström and Muranen, 2025). Additionally, these cytokines interact with immune cells, forming an immune escape microenvironment (Baldassarri et al., 2024). Xu et al. showed that CMA cells promote EMT in tumor cells by autocrine secretion of CCL5, which interacts with CCR5 receptors, enhancing tumor migration and invasion. Additionally, CCL5 induces macrophage polarization toward the M2 phenotype and inhibits T-cell infiltration, further reinforcing immune escape mechanisms (Xu et al., 2023).

However, despite the significant insights provided by organoid models into the complex dynamic interactions between bone tumors and their microenvironment, several limitations remain. These models primarily simulate local TME changes, restricting their ability to capture the complexity of systemic immune. Additionally, organoid-based research often emphasizes local immune evasion mechanisms, overlooking the broader immunological dynamics that influence tumor progression and therapeutic outcomes. Therefore, future studies need to integrate organoid models with a broader immunological perspective to gain a more comprehensive understanding of tumor-matrix interaction mechanisms.

### 4.2 High-throughput screening platforms

The integration of high-throughput screening platforms with organoid technology is gradually revolutionizing traditional drug development processes. This has enabled researchers to efficiently test large numbers of drugs or drug combinations and evaluate their efficacy in various disease models. For instance, Shihabi utilized CMA organoid models to rapidly screen hundreds of drugs, successfully identifying several effective candidates, including PI3K/mTOR inhibitors such as sapanisertib and vistusertib, EGFR-targeted drug gefitinib, and JAK2/STAT3 inhibitors like fedratinib (Al Shihabi et al., 2022). Similarly, researchers have used OS organoids to screen a range of FDA-approved kinase inhibitors, rapidly identifying inhibitors targeting the PI3K/AKT/mTOR and MAPK pathways, which showed significant efficacy in co-suppressing OS growth (Zhang et al., 2024a).

Bone tumors frequently exhibit substantial resistance to chemotherapy drugs. Therefore, evaluating organoid responses to specific drugs allows for the identification and exclusion of ineffective or highly toxic drugs, thereby optimizing treatment selection and minimizing adverse effects. For example, Wang et al., they discovered that tumor stem cell resistant subpopulations, such as CD117^+^, showed poor response to chemotherapy drugs. However, the administration of SKP2 inhibitors effectively reduced the proportion of the CD117^+^ subpopulation, thereby restoring the sensitivity of tumor cells to chemotherapy and enhancing treatment efficacy. The SKP2 inhibitor C1 sensitized traditional chemotherapy drugs by increasing p27 levels, delaying the OS cell cycle, and promoting E2F1 conversion to an apoptotic state (Wang et al., 2024). Additionally, in studies evaluating combination therapies for PCa BoM, ruxolitinib, effectively inhibited the IL-6/JAK/STAT3 signaling pathway in IL-6-driven models, thereby restoring sensitivity to anti-hormone therapy. In contrast, in IL-6-independent models, specific kinase inhibitors targeting the ERK and PI3K/Akt pathways successfully overcame drug resistance (Ji et al., 2023).

Furthermore, organoid models demonstrate significant advantages in addressing the limitations of existing treatments. For example, compound S6 can inhibit tumor cell growth by disrupting the SOST-STAT3 protein interaction. Compared to the conventional treatments, S6 does not target the SOST loop2 region, effectively avoiding cardiovascular toxicity risks posed by conventional therapies like romosozumab which target the SOST-loop2 region (Sun et al., 2022). These finding provides a strong scientific basis for the development of novel drugs.

### 4.3 Bone defect repair

In regenerative medicine, organoids technology presents promising therapeutic potential and reparative strategies for bone tumor patients. The invasive nature of bone tumors often results in severe damage to bone tissue structure and functional impairment. While conventional treatments primarily focus on tumor excision, effective post-surgical bone regeneration remains a significant challenge. In this regard, bone tumor organoids combined with biomimetic scaffold materials have emerged as a novel approach to promoting bone tissue repair and functional restoration.

Huang et al. constructed PCL@Cu-HHTP composite scaffolds that could efficiently release Cu^2+^ thereby induce the production of reactive oxygen species, simulating oxidative stress conditions observed in the TME. The hydrophilic and bioactive surface of the scaffold promoted the adhesion and proliferation of BMSCs. The scaffold’s porosity and mechanical properties created a support environment for the OS organoid culture. Given its good photothermal effects, the scaffold accelerated chemical reactions that lead to the decomposition of OS cells, achieving promising therapeutic effects (Huang B. et al., 2024). In the study Han et al., a DNA hydrogel combined with tFNA and Aptamer02, was designed, and found to inhibit the Hippo signaling pathway and activate the VEGFR signaling pathway, to promote osteogenesis and angiogenesis of the bone. The proposed hydrogel not only exhibited excellent editability and long-term release functions, but also maintained good mechanical stability when administered with PCL scaffolds, providing mechanical support and cell attachment sites for bone repair (Han et al., 2024).

The organoids have provided suitable platforms for exploring the osteoclastic mechanisms of bone tumors. A previous study used organoid investigated the expression of bone remodeling cell subpopulations and genes related to osteoblast inhibition, such as *SFRP1*, *NPPC*, and *RACK1* in organoids (Xiong et al., 2024). Their results provided foundational data that will guide future application of regenerative medicine to improve bone metabolism.

## 5 Cutting-edge technologies advances

### 5.1 CRISPR-Cas9 technology

CRISPR-Cas9 technology provides a valuable tool for gene-editing that has improved cancer research. Using this technology, gene mutations and knockouts have been established in patient-derived organoids. Therefore, the technology only improves the genetic fidelity of bone tumor organoids but also ensures the stability and representativeness of their genomes. Following CRISPR-Cas9-mediated gene editing, the organoids are cultured to examine whether the genetic modifications remain stable across multiple cell generations.

In bone tumor research, the common genetic mutations investigated include mutations in tumor suppressor genes like *p53* or oncogenes like *Ras*. The CRISPR-Cas9 has been used to create *p53* knockout OS cell lines, revealing that, although the p53 mutation can inhibit tumor cell growth, it cannot completely prevent tumor invasion and metastasis (Shimizu et al., 2022). To improve accuracy and stability of gene editing, high-throughput genomic sequencing techniques are adopted to analyze the mutations introduced by CRISPR-Cas9 and assess off-target effects. In this approach, genetically stable clones are selected through single-cell cloning, which ensures the genetic consistency of the organoids. For example, Gerardo-Ramírez successfully created CD44 knockout OS organoids using CRISPR/Cas9 technology and validated its genetic stability post-editing through PCR amplification, Sanger sequencing, and RNA-seq (Gerardo-Ramírez et al., 2022). Elsewhere, Zhang et al. explored the doxorubicin-induced sensitivity using CRISPR technology, identifying that the kinase PRKDC, which was validated through cell experiments and mouse xenograft models. They also tested the combination therapy of PRKDC inhibitor AZD7648 with doxorubicin using organoids, creating a new experimental model and therapeutic strategy for precision medicine and bone tumor treatment (Zhang et al., 2024a).

### 5.2 Organ-on-a-chip

Organ-on-a-chip (OoC) technology is a novel technology based on microfluidic chips and cell culture techniques. The OoC allows precise control of fluid flow in tiny channels, thereby simulating the physiological environment of human organs or tissues. Compared to traditional laboratory culture methods, OoC is more effective in recreating the blood flow characteristics and dynamic changes in the TME, providing more accurate biological models. For example, Ji et al. designed microfluidic channels with an H-type vascular structure and successfully recreated changes in the vascular microenvironment during bone metastasis by implanting HUVECs into the chip. The chip not only enhanced nutrient exchange and fluid transmission for cell culture, but also allowed dynamic monitoring of the interaction between LCa cells, BMSCs, and osteoclasts (Ji et al., 2023). In a study by Lu, a multi-level bionic OoC was constructed with an integrated recirculation system, which successfully simulated the functionality of the bone marrow niche, providing a new platform for bone tumor research (Lu et al., 2024). Recent researches have demonstrated that OoC not only simulates the microenvironment around human tumor blood vessels but also promotes the formation of vascularized OS organoids. This multi-channel design provides a platform for the co-culture of tumor cells and microvascular systems, successfully replicating the natural heterogeneous angiogenesis features seen in clinical tumor tissues, effectively simulating the OS TME (Du et al., 2025).

Besides tumor models, the OoC technology has enabled the creation of new methods for drug screening. The microfluidic chips have been used to culture 3D spheroids of ES cells, creating an efficient platform for high-throughput screening of drug responses (Fevre et al., 2023). Mechanistically, the two chips usually synergize: one facilitates the culture of bone tumor cells and formation of organoids, and the other for encapsulation of drug solutions and introducing them into the organoids for testing (Kheiri et al., 2024).

Furthermore, the OoC technology can potentially enhance early diagnosis of OS. Specifically, microfluidic technology has been employed to isolate exosomes from human plasma and organoid cell culture media. The surface-enhanced Raman scattering technology was then applied to analyze the biomarkers on the surface of exosomes, thereby improving early diagnosis of OS (Han et al., 2022).

### 5.3 Artificial intelligence

The integration of artificial intelligence (AI) with organoid research has significantly enhanced data processing and analysis efficiency. Through high-throughput data screening and deep learning algorithms, AI allows researchers to efficiently perform drug screening and construct predictive tools for various treatments (Zhou et al., 2022). This technology is particularly beneficial in the field of tumor behavior analysis and image recognition, owing to its potential to automate the processing of large amounts of 3D imaging data, making it ideal for discovering the multi-dimensional features of organoids during growth and monitoring their size, morphological changes, and biological status (e.g., activity, death, differentiation). Using machine learning, 3D structural images can be analyzed to obtain important features to accurately classify and quantify iPSCs into embryonic-like structures. This technology mirrors the analysis of morphological changes, proliferation patterns, and treatment feedback of tumor cells in three-dimensional cultures (Guo et al., 2021). Moreover, AI can perform large-scale data mining and pattern recognition allowing analysis of growth curves of tumor organoids, identifying growth characteristics and patient-specific features. For instance, Matthews et al. developed OrganoID, an image analysis platform that employs convolutional neural networks to classify and track various tumor organoid types at the pixel level, enabling quantitative assessment of their morphological characteristics (Matthews et al., 2022). This innovative approach eliminates the reliance on genetic markers or harmful dyes for precise measurements and facilitates automated high-throughput experimental analysis.

In recent years, the AI technology is widely being applied in research into organoids for various organs, such as the pancreas, lung, and colon, but its application in bone or bone tumor organoids is relatively limited. Some studies have shown that AI-assisted quantum dots combined with bone organoids can examine the structure of bone cells and tissues. The quantum dots allow precise tracking of drug distribution in bone tissue and identification of biomarkers associated with bone tumors owing to their fluorescent properties (Ding et al., 2024). In addition, AI models can predict the prognosis of ameloblastoma and analyze tumor cell interactions, thus uncover potential therapeutic targets like FOSL1, BRD4, EZH2, and Wnt signaling pathways (Liu Y. et al., 2025).

However, the application of AI in bone tumor organoids has not improved significantly due to the following factors: First, the imaging features of different tumors vary, making it difficult for AI models to accurately differentiate them. Second, due to the bone-invasive nature of bone tumors and the complexity of their microenvironments, culture of bone tumor organoids is challenging, and there is lack of sufficient samples for the training of AI models. Third, the full functionality of AI models is not well understood, and the reasoning processes associated with the algorithms are unclear, decreasing trust in AI technology, especially when dealing with significant health issues such as bone tumors.

## 6 Challenges and limitations

Currently, there is no standardized process for establishing bone tumor organoids. Moreover, some of the available methods are not internationally recognized, making it difficult to compare and reproduce them. Therefore, future studies should aim to promote the establishment of standardized culture conditions or develop universal organoid models to facilitate international research and reproducibility of research findings. Another limitation is the vascularization process for organoids. Notably, the growth of bone tissue and tumors in the human body is highly dependent on vascular networks that provide nutrient and waste exchange. Although advancements have been made in the co-culturing of endothelial cells or leveraging microfluidic chip technologies, the integration of 3D culture platforms and identification of the intricate cellular interactions are major challenges that need to be addressed.

Furthermore, clinical translation of existing models faces significant challenges as the models fail to simulate the dynamic processes such as the imbalance between osteolysis and osteogenesis, tumor immune escape, and cellular resistance. Although current bone tumor organoids can reflect tumor characteristics at a microscopic scale, their small size and limited blood supply renders them unable to simulate tumor growth and metastasis in the human body. Although organoid technology can help researchers to establish personalized tumor models, their production is costly and time-consuming (Sanchez et al., 2024), which limit their widespread application in clinical practice.

Moreover, there are limitations associated with ethical and regulatory. The process of obtaining organoids from source organs may involve several ethical challenges in terms of patient privacy, the balance between free will and research needs, and practices such as the use of human embryonic stem cells in organoid cultures, which delays their use. There are also challenges regarding data misuse, ensure safety, and quality control in the use of organoids. Depending on the legal framework, organoids can be categorized as drugs, medical devices, or experimental research materials (Reyes et al., 2024), which makes their regulation complex and challenging. Establishing international standard procedures and ethics of regulation can help reduce unnecessary variations in organoid culture, preservation, and transfer processes.

## 7 Conclusion and prospect

In recent years, significant progress has been made in bone tumor organoid research, However, some limitations, particularly in areas such as simulating the real TME, constructing vascular structures, and modeling immune responses remain, which need to be resolved in further studies. Future research should aim to address these bottlenecks, especially the issues of scaling up organoid production, quality control, and technical standardization.

Overall, this review demonstrates that bone tumor organoids have important clinical value. This review highlights the establishment methods and applications of bone tumor organoids, outlines the key directions for future research, uncovering their role in drug development and regenerative medicine. In future, targeted research strategies are needed to expedite the translation of bone tumor organoids into clinical.

## Glossary

2D: Two-dimensional
3D: Three-dimensional
AI: Artificial intelligence
ALI: Air-liquid interface
BCa: Breast cancer
BMSCs: Bone marrow-derived mesenchymal stem cells
BoM: Bone metastasis
CAR-T: Chimeric antigen receptor T-cell
CMA: Chordoma
CS: Chondrosarcoma
eBM: Engineered bone marrow
ECM: Extracellular matrix
EGF: Epidermal growth factor
EMT: Epithelial-mesenchymal transition
EPC: Endothelial progenitor cell
ES: Ewing sarcoma
HUVEC: Human umbilical vein endothelial cell
IF: Immunofluorescence
iPSC: Induced pluripotent stem cell
LCa: Lung cancer
MAPK: Mitogen-activated protein kinase
M-CSF: Macrophage colony-stimulating factor
MGCTB: Malignant giant cell tumor of bone
MM: Multiple myeloma
MSC: Mesenchymal stem cell
OoC: Organ-on-a-chip
OS: Osteosarcoma
PCa: Prostate cancer
PCL: Polycaprolactone
PD: Patient-derived
PLGA: Poly (lactic-co-glycolic acid)
RANKL: Receptor activator of nuclear factor-κB ligand
TGF-β: Transforming growth factor beta
TME: Tumor microenvironment
